# Experimental rewilding may restore abandoned wood-pastures if policy allows

**DOI:** 10.1007/s13280-020-01320-0

**Published:** 2020-03-09

**Authors:** Pablo Garrido, Lars Edenius, Grzegorz Mikusiński, Anna Skarin, Anna Jansson, Carl-Gustaf Thulin

**Affiliations:** 1 School for Forest Management, Faculty of Forest Sciences, Swedish University of Agricultural Sciences, 739 21 Skinnskatteberg, Sweden; 2 Department of Wildlife, Fish, and Environmental Studies, Swedish University of Agricultural Sciences, 901 83 Umeå, Sweden; 3 Department of Anatomy, Physiology and Biochemistry, Swedish University of Agricultural Sciences, 750 07 Uppsala, Sweden; 4 Department of Ecology, Swedish University of Agricultural Sciences, 730 91 Riddarhyttan, Sweden; 5 Department of Animal Nutrition and Management, Swedish University of Agricultural Sciences, 750 07 Uppsala, Sweden

**Keywords:** Ecological replacement species, Horse browsing, Paradigm shift, Political constrain, Rewilding, Wood-pasture restoration

## Abstract

Large herbivores play key roles in terrestrial ecosystems. Continuous defaunation processes have produced cascade effects on plant community composition, vegetation structure, and even climate. Wood-pastures were created by traditional management practices that have maintained open structures and biodiversity for millennia. In Europe, despite the broad recognition of their biological importance, such landscapes are declining due to land-use changes. This calls for finding urgent solutions for wood-pasture conservation. To test whether introducing an ecological replacement of an extinct wild horse could have positive effects on wood-pasture restoration, we designed a 3-year rewilding experiment. Horses created a more open wood-pasture structure by browsing on seedlings and saplings, affected tree composition via selective browsing and controlled the colonization of woody vegetation in grassland-dominated areas. Thus, rewilding could be a potential avenue for wood-pasture restoration and biodiversity conservation. However, such benefits may not materialize without a necessary paradigm and political shift.

**Electronic supplementary material:**

The online version of this article (10.1007/s13280-020-01320-0) contains supplementary material, which is available to authorized users.

## Introduction

Large herbivores play a key role in the functioning of many terrestrial ecosystems (Doughty 2017). Due to their large body size, they have a disproportionate effect on ecosystems (Owen-Smith 1987), including ecosystem engineering functions (Haynes 2012). They affect the vegetation structure through feeding and seed dispersal, nutrient cycling, and even climate (Bakker et al. 2016; Cromsigt et al. 2018b). Diverse large herbivore communities usually promote more open landscape conditions (Bakker et al. 2016).

Since the offset of the global human expansion, prehistoric megaherbivore faunas have been largely depleted (Sandom et al. 2014), which led to the progressive simplification of megafaunas on ecosystems, with cascade effects on plant community composition, vegetation structure, and fire regimes (Gill 2014). Such defaunation processes have also produced ecological state shifts in different biomes (Barnosky et al. 2016). For instance the disappearance of numerous megaherbivores in Siberia resulted in dramatic changes from a mammoth-rich steppe habitat towards the tundra and boreal forest ecosystems of today (Zimov et al. 2012).

Large herbivores have historically been considered detrimental to forests ecosystems due to negative effects on tree regeneration and recruitment (Gill 2006). However, such an argument has been challenged based on ecological and historical research (Vera 2000). Vera (2000) stated that wood-pasture mosaics and the regeneration of light-demanding trees such as oaks (*Quercus* spp.) might be connected to the presence of large herbivores including livestock. Indeed, large herbivores were present in primeval forest ecosystems (Vera et al. 2006) as aurochs (*Bos taurus* L.) and tarpans (*Equus ferus* L.) that were later replaced by their domestic forms cattle and horses (Vera et al. 2006). These large herbivore grazers are fundamental in wood-pasture formation and dynamics (Vera et al. 2006). Oldén et al. (2017) have recently investigated how grazing and abandonment determined different tree dynamics in boreal wood-pastures in Finland, highlighting the importance of resume livestock grazing for wood-pasture formation and development. Bocherens (2018) recently postulate that some of the megaherbivore functions in ecosystems were replaced by human agriculturalists through animal and plant domestication, and agriculture and husbandry practices. Thus human traditional multi-functional management practices, including grazing with livestock, have maintained wood-pastures and cultural landscapes for millennia (Blondel 2006), resulting in highly diverse habitats that uphold important ecological and cultural values (Plieninger et al. 2015). In Sweden as in the rest of Europe, the abandonment of these traditional management practices clearly threatens the conservation value of wood-pastures and associated biodiversity (Bergmeier and Roellig 2014; Plieninger et al. 2015; Garrido et al. 2017). This calls for finding plausible alternatives for restoring these declining ecosystems (Wright et al. 2012). Such interventions may have a significant effect on entire landscapes currently experiencing high rate of agricultural land abandonment (Donlan et al. 2006; Navarro and Pereira 2015).

Trophic rewilding is a restoration strategy focused on reintroducing missing animal taxa to promote self-regulating biodiverse ecosystems by restoring trophic top-down interactions and associated trophic cascades (Svenning et al. 2016; Torres et al. 2018). To enrich a browser-dominated herbivore community by introducing an ecologically functional substitute of an extinct large herbivore grazer, may therefore mitigate current biodiversity declines and restore abandoned wood-pastures. This may occur by the above-mentioned replacement of former human management practices by analogue large herbivore functions (Pedersen et al. 2019). Currently, there is a severe lack of empirical rewilding experiments (Svenning et al. 2016) and is therefore urgent to design scientific experiments to advance rewilding science. Such evidence-based knowledge may inform future biodiversity-oriented management programs, restoration ecology and conservation. Indeed, novel conceptual frameworks are just being developed to design and evaluate different rewilding approaches and integrated conservation outcomes (Torres et al. 2018; Pedersen et al. 2019; Perino et al. 2019). Horses are key candidate species for rewilding due to the widespread occurrence of suitable climates and habitats within their historical distributional range, their important ecological functions as grazers, as well as the extensive knowledge on their ecology, behavior, and management (Naundrup and Svenning 2015). Although primarily grazers, horses might include a significant proportion of woody plants in their diet (see Gill 2006). To test whether the introduction of a functional substitute of an extinct wild horse could have positive effects on wood-pasture restoration, we designed a 3-year rewilding experiment where an endangered Swedish horse breed, *Equus ferus* L.(Gotland Russ), was introduced in three 10 ha enclosure replicates. We investigated the cumulative effect of horse browsing on the vegetation structure and composition, and quantified (1) browsing pressure, (2) tree consumption, and (3) tree selectivity estimates for eight common tree species. This novel knowledge is crucial for implementing future rewilding experiments, biodiversity conservation, and wood-pasture restoration programs. However, this may not materialize without a necessary paradigm and political shift to promote rewilding interventions.

## Materials and methods

### Study area and experimental design

**Fig. 1:**
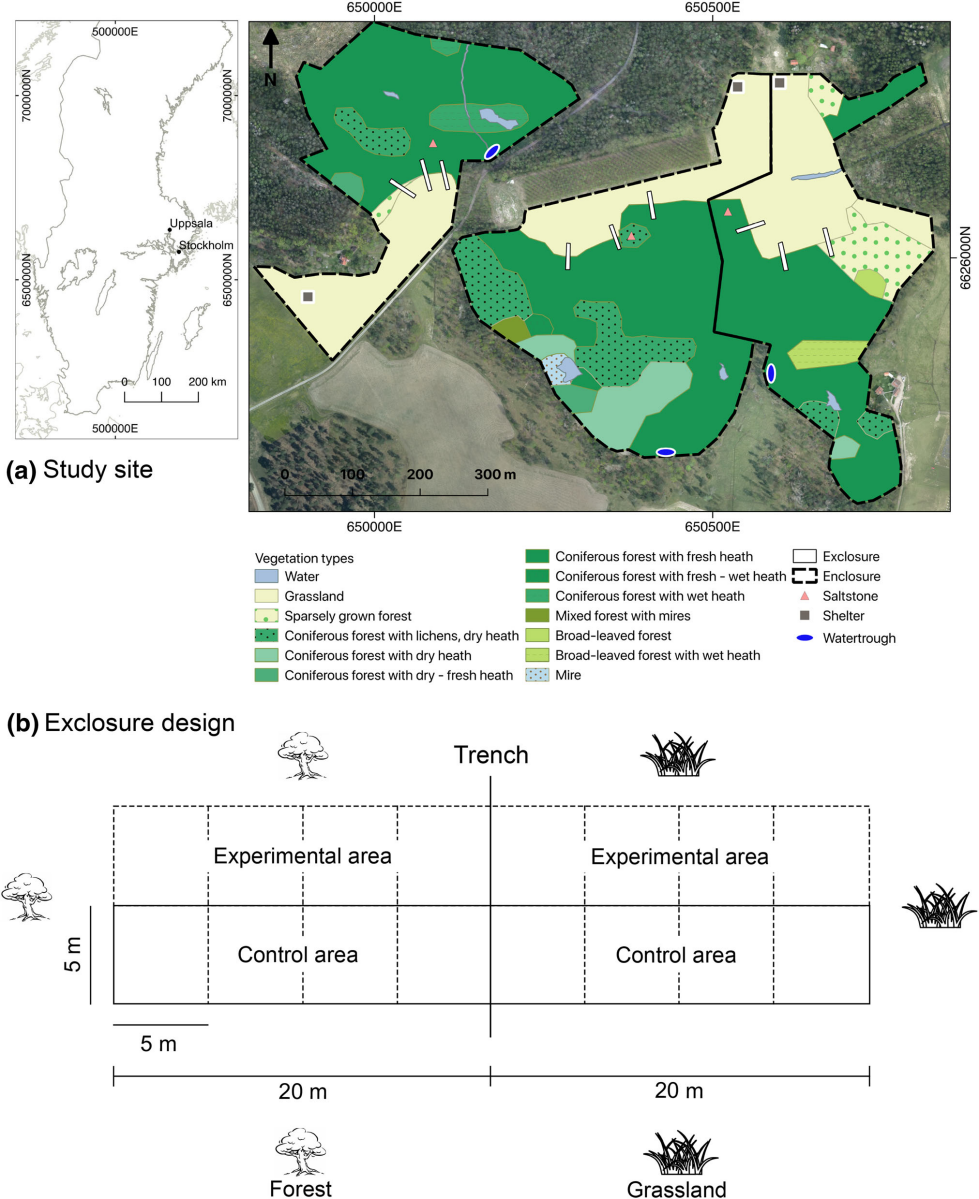
**a** Study area and experimental design. Exclosures represent control plots where herbivory was excluded. Their location on the map is approximate (see “Materials and methods” for details). The enclosures were considered the experimental area where horses were introduced. **b** Vegetation surveys in the experimental areas were performed parallel to exclosures (controls; see details in **b**)

The study was performed at Krusenberg (Fig. 1a), an estate of 842 ha located 17 km south of Uppsala (59° 44′ N 17° 40′ E; Sweden), owned and managed by the Swedish University of Agricultural Sciences. The property contains 204 ha of agricultural land, 72 ha of pasture and grasslands, 510 ha of forest, and 46 ha correspond to other land uses (Päiviö 2008). Here a 3-year experiment was conducted at three different 10 ha wood-pasture enclosures (Fig. 1a), where four free-ranging 1-year-old horse stallions were released per enclosure in May 2014 (Gotland Russ; average stocking rate 0.35 horse/ha; average body mass 250 kg/horse). Because of the feral history of the breed, it may have retained a sufficient rustic character (e.g., energy retaining characteristics and feeding behavior) to serve as an ecologically functional substitute of extinct wild horses, while fostering the conservation of a critically endangered national breed; liability commissioned by FAO (First report on the state of the world’s animal genetic resources), according to which Sweden is obliged to preserve the breed for future generations. Horses were kept without supplemental feeding (however, with salt blocks including trace minerals) until September 2016 (experiment approved by Uppsala Animal Welfare Committee, Ethical Approval Number C28/14). Body condition was monitored daily according to Henneke et al. (1983) and horses that scored < 4 (4 equals “moderately thin”) were temporarily removed from the enclosures and offered additional pasture. During the study period, four individuals were temporarily removed due to low body condition in late winter. They were additionally dewormed when necessary using predefined indicators (Tydén et al. 2019) and were provided with artificial shelter and water in troughs (one per enclosure according to national regulations). From January 2016, one horse was excluded from the study due to injury. Each enclosure consisted of 2.9 ha ± 1.9 (SD) of grassland (average productivity 2300 kg dry matter/ha; Ringmark pers. comm.) and 8.0 ha ± 1.8 (SD) of forest (see legend in Fig. 1a for detail description of forest characteristics per enclosure). The experimental area is defined as a wood-pasture mosaic where forest dominated areas are interspersed with grasslands. Along the edge zone between forest and grasslands in each enclosure, three rectangular 40 × 5 m (20 m into the forest and 20 m into the grassland) exclosures (control areas) were placed in May 2014. Exclosures and equally sized experimental areas were divided into eight squared 5 × 5 m plots. In each paired 5 × 5 m control–experimental plot, woody vegetation was surveyed (Fig. 1b). Browsed/grazed areas resembled the restoration of wood-pastures through browsing and grazing by a richer herbivore community (adding a grazer into a browser-dominated community). Exclosures represented land abandonment conditions (cessation of traditional summer grazing and mowing). Sympatric large herbivore browsers in the study area include roe deer *Capreolus capreolus* L. and moose *Alces alces* L. The number of surveyed plots amounted to 144. From 2004 to 2014, the experimental area was abandoned, pasturelands were not tilled, and were only occasionally harvested and/or grazed (including the forest) by cattle (Ryberg, pers.comm.). Due to the observed woody encroachment of grasslands and abundant regeneration of forests, such occasional use was considered to have negligible effects. The area is located within the hemiboreal vegetation zone (Ahti et al. 1968). Mean temperature in the study period ranged from − 4.8 °C (± 6.5 SD) in January to 17.5 °C (± 4.1 SD) in July. Rainfall ranged from 65 to 123 mm in July during the same period.

### Vegetation survey

For each paired 5 × 5 m control–experimental plot, the total number of trees > 20 cm was counted in September 2016 and identified to species (except for *Salix* spp.). Tree height (cm), diameter (mm), total number of twigs, and total number of browsed twigs were recorded per tree. Diameter of trees higher than 3 m was measured at breast high (DBH = 1.30 m), 5 cm from the ground otherwise. Both old and fresh bites were recorded since we wanted to estimate the cumulative effect of browsing on the vegetation structure and composition after the 3-year experimental time. Browsing pressure was investigated up to 3 m height, i.e., the maximum browsing height for moose (Bergström et al. 1995) and assumed to be the maximum reachable height for the horses (height at withers 115–130 cm).

### Statistical analysis

#### Effects on forest structure

We tested whether tree height/diameter ratios (as a measure of vegetation structural change) were significantly different in experimental and control conditions. This was separately investigated in forest and grassland subplots. We used General linear mixed models (GLMM) with a nested random structure term (i.e., plot within exclosure and enclosure) fitted to a Gaussian distribution (package *nlme*; Pinheiro et al. 2017).

#### Browsing pressure and tree selection

In order to calculate the relative proportion of tree species exposed to browsing, a general model for selectivity was applied (Chesson 1978). This consisted in three different parameters organized as follows:

1 $$u_{i} = \frac{{v_{i} a_{i} }}{{\mathop \sum \nolimits_{i = 1}^{I} v_{i} a_{i} }},$$

where *u*_*i*_ is the relative proportion of utilized food item *i*, based on browsing pressure estimates per individual tree species; *v* corresponds to a selectivity parameter; *a* refers to the proportion of available food item *i*, computed as the number of trees of species *i* divided by the total number of trees per plot; and *I* represents the total number of tree species considered, including *Pinus sylvestris* L. (Scots pine), *Betula pendula* Roth (silver birch), *Fraxinus excelsior* L. (European ash), *Populus tremula* L. (aspen), *Prunus spinosa* L. (blackthorn), *Quercus robur* L. (pedunculate oak), *Salix* spp. (sallow), and *Sorbus aucuparia* L. (rowan). Tree species with low occurrences and on grassland-dominated areas^1^ were excluded (see Table S1). *Picea abies* (L.) H. Karst. (Norway spruce) was also excluded due to observed avoidance (see complete species list in Supplementary Material; Table S1). Forage availability was calculated based on the selected eight tree species present per plot. Trees up to 5 m were included in the analysis; higher trees were excluded due to limited effect of browsing.

To find the selectivity parameter *v* for each tree species, Eq. 1 can be re-organized so that

2 $$v_{i} = \frac{{u_{i} }}{{a_{i} \sum\nolimits_{i = 1}^{I} {(u_{i} /a_{i} )} }}$$

since the relative proportion of utilized tree species *u* sum to 1. One of the strengths of the model is the possibility to compare selectivity parameters for different tree species. However, this index is not normally distributed and therefore a log transformation is suggested (Aitchison 1986).

3 $$x_{i} = \ln \left( {\frac{{v_{i} }}{{v_{0} }}} \right)$$

where *v*_*i*_ represents the selectivity parameter for the tree species *i* and *v*_0_ denotes the selectivity parameter of a reference species. Therefore a preference index can be calculated in relation to a species of reference, i.e., Scots pine in this case because of lowest relative browsing pressure. For analyses and plotting, R version 3.3.3 (R Core Team 2017) was used.

## Results

In total, 1226 individual trees were counted in forest subplots and 291 in grassland subplots (Table S1). The number of trees was higher in controls compared to experimental plots (*t* test; *t* = − 12.22, df = 1057.4, *p* value < 0.001; Figs. S1, S2).

### The effect of browsing on forest structure

**Fig. 2:**
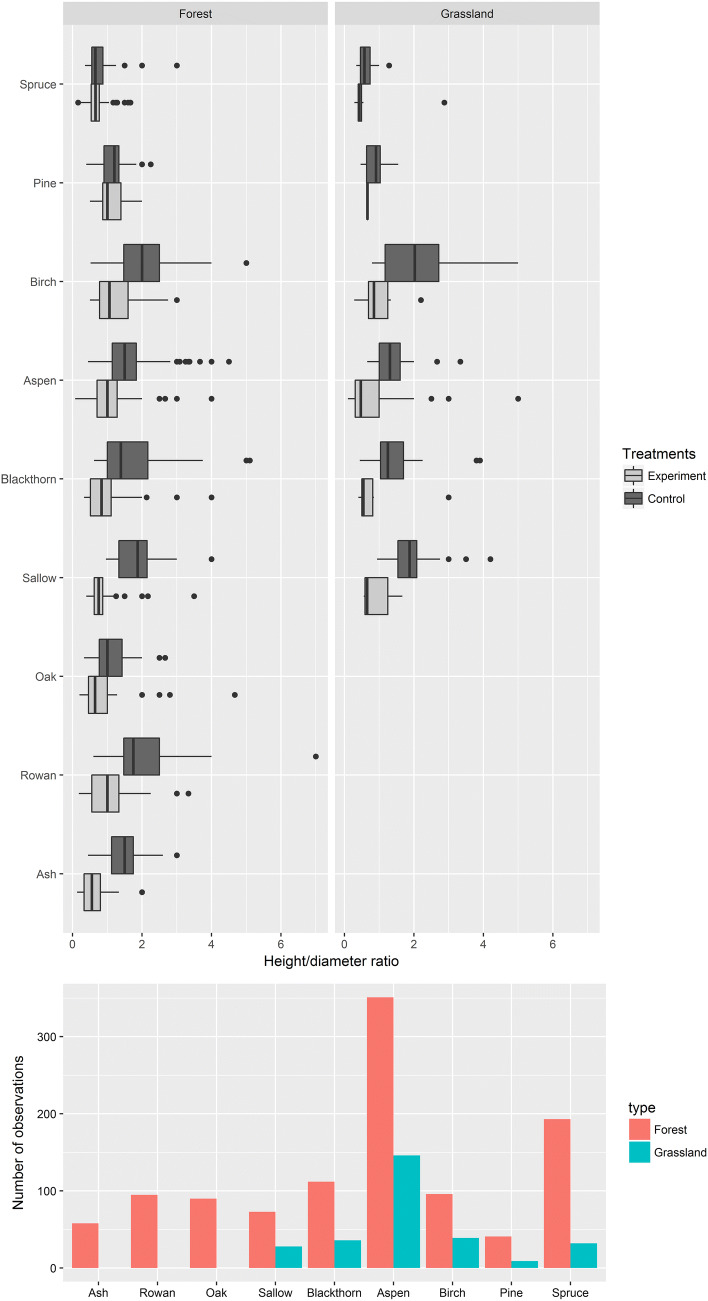
Height/diameter ratio for trees in experimental and control plots. This ratio was utilized as proxy to elucidate horse browsing effect on vegetation structure

A total of 15 tree species were recorded (Table S1). Horse browsing significantly reduced height/diameter ratios for ash, rowan, sallow, blackthorn, aspen, and birch in forest subplots (see Table 1; Fig. 2). For oak, pine, and Norway spruce, no effect was detected (Table 1). In grassland subplots, reductions were detected for birch, aspen, and sallow, but not for Norway spruce and blackthorn (see Table 1).

**Table 1:** Results of modeling height/diameter ratios for woody vegetation in relation to treatment. General linear mixed effect models (GLMM) fitted to a Gaussian distribution were used. *β* regression coefficient estimate, *SE* standard error, *DF* degrees of freedom

	*β*	SE	DF	*t* value	*p* value
**Forest subplots**					
*Scots pine*					
Intercept	1.56	0.30	33	3.86	< 0.01
Control	− 0.09	1.18	33	− 0.48	0.63
*Norway spruce*					
Intercept	0.71	0.07	181	10.11	< 0.01
Control	0.08	0.05	181	1.78	0.08
*Silver birch*					
Intercept	1.44	0.21	85	6.96	< 0.01
Control	0.60	0.19	85	3.21	< 0.01
*Aspen*					
Intercept	1.34	0.28	341	4.78	< 0.01
Control	0.48	0.07	341	7.24	< 0.01
*Blackthorn*					
Intercept	0.98	0.25	108	3.88	< 0.01
Control	0.75	0.17	108	4.53	< 0.01
*Sallow*					
Intercept	1.05	0.16	65	6.49	< 0.01
Control	0.86	0.15	65	5.74	< 0.01
*Pedunculate oak*					
Intercept	1.05	0.24	80	4.32	< 0.01
Control	0.15	0.17	80	0.90	0.37
*Rowan*					
Intercept	1.14	0.14	85	8.02	< 0.01
Control	0.89	0.19	85	4.77	< 0.01
*European ash*					
Intercept	0.64	0.20	54	3.23	< 0.01
Control	0.84	0.13	54	6.39	< 0.01
**Grassland subplots**					
*Norway spruce*					
Intercept	0.67	0.26	28	2.52	0.02
Control	− 0.04	0.18	28	− 0.23	0.82
*Silver birch*					
Intercept	1.11	0.31	31	3.56	< 0.01
Control	0.79	0.35	31	2.28	0.03
*Aspen*					
Intercept	1.19	0.23	141	5.12	< 0.01
Control	0.57	0.10	141	5.48	< 0.01
*Blackthorn*					
Intercept	0.87	0.44	32	1.96	0.06
Control	0.58	0.31	32	1.89	0.07
*Sallow*					
Intercept	0.97	0.31	22	3.11	< 0.01
Control	0.99	0.35	22	2.84	< 0.01

### Browsing pressure and tree selectivity

Trees were not consumed proportionally to availability (see Table 2). Ash was the most browsed tree species with an average browsing pressure of 0.71, i.e., more than 70% of all available twigs per tree were browsed. In contrast, pine was the least browsed (Norway spruce excluded) with an average browsing pressure of around 0.19 (Table 2). This resulted in a selectivity index for ash 4.0 times higher compared to pine, for rowan and oak around 3.0 times higher, 2.5 for sallow, blackthorn and aspen less than 2.0, and birch was the relatively least preferred species in relation to pine with 1.4.

**Table 2:** Mean values ± SD for tree species availability and horse browsing pressure estimates. Consumption, selectivity parameters, and relative preference estimates are also shown per tree species

	European ash	Rowan	Pedunculate oak	Sallow	Blackthorn	Aspen	Silver birch	Scots pine
Tree species availability^a^	0.11 ± 0.12	0.13 ± 0.07	0.11 ± 0.11	0.08 ± 0.10	0.13 ± 0.13	0.31 ± 0.17	0.10 ± 0.08	0.02 ± 0.04
Browsing pressure^b^	0.71 ± 0.10	0.55 ± 0.12	0.53 ± 0.11	0.47 ± 0.13	0.36 ± 0.14	0.35 ± 0.14	0.27 ± 0.13	0.19 ± 0.11
Consumption^c^	0.08 ± 0.02	0.07 ± 0.02	0.06 ± 0.02	0.04 ± 0.03	0.05 ± 0.04	0.11 ± 0.05	0.03 ± 0.02	0.004 ± 0.010
Selectivity parameter^d^	0.21	0.16	0.15	0.14	0.10	0.10	0.08	0.05
Relative preference^e^	3.73	2.89	2.79	2.49	1.89	1.86	1.40	****

****Represents the reference species. ^a^Tree species (forage) availability was estimated as the number of focal tree species divided by the total number of species recorded in experimental plots. Species with low occurrences and Norway spruce were excluded from the calculations. ^b^Browsing pressure was obtained as the ratio between the number of browsed twigs divided by the total number of twigs up to 3 m per selected tree species. ^c^Consumption was computed as the product of the previous to values. ^d^The selectivity parameter per tree species was calculated based on Eq. 2 (see “Statistical analysis”). ^e^The relative preference index (log-transformed) was computed using Eq. 3 with Scots pine as reference species, i.e., the denominator in Eq. 3

## Discussion

To our knowledge, we present the first empirical study on the potential of rewilding with horses (as an ecologically functional replacement species of extinct wild horses) to restore wood-pastures, and provide the first quantitative estimates of browsing pressure and tree selection for eight common tree species for temperate ecosystems. This novel knowledge is crucial to inform future conservation-oriented management programs.

Traditionally, cattle and more locally sheep have been grazing wood-pastures in Europe. However, such practices were normally restricted in time (season) or animals were artificially fed in winter which calls for caution when compared to our results (Bernes et al. 2018; Öllerer et al. 2019). Nonetheless livestock grazing generally decrease the abundance of understory vegetation (Bernes et al. 2018). Livestock grazing also increase habitat heterogeneity and affect forest structure and composition (Öllerer et al. 2019). Long-term observational studies additionally confirm the above-mentioned effects of cattle grazing in the UK (Mountford et al. 1999; Harmer et al. 2001). However, only from 2001 to 2012, almost eight million hectares of farmlands have been abandoned in Europe, mainly due to biophysical or accessibility constrains (Estel et al. 2015; Lasanta et al. 2017). Similarly in Sweden, traditional management practices that maintained wood-pastures and semi-natural grasslands are threatened as a result of land-use changes (Garrido et al. 2017). This resulted in either agricultural intensification or abandonment of marginal lands. Abandoned wood-pastures develop secondary woodlands (normally Norway spruce in Sweden) which reduces biodiversity (Paltto et al. 2011). In boreal Finland, Oldén et al. (2017) showed that spruce regeneration was abundant in all kinds of abandoned wood-pastures (birch-, pine-, spruce- and broadleaved-mixed dominated wood-pastures) and regeneration was only reduced by removal or by increasing canopy openness. In our experimental study spruce was avoided. In this regard, Oldén et al. (2017) concluded that sprucification is a major problem in wood-pastures due to resulting landscape homogenization and vegetation compositional changes towards spruce-dominated wood-pastures and suggested spruce regeneration removal to benefit rare plant and insect species characteristic of semi-open woodlands (Oldén et al. 2017). A recent study reported that 66% of the most valuable oak wood-pasture habitats in Östergötland (Sweden) were abandoned (with subsequent secondary woodland development) and grazing could not be resumed due to lack of farmers and livestock (Garrido et al. 2017). This is a common pattern also in Europe (Bergmeier et al. 2010; Bergmeier and Roellig 2014; Plieninger et al. 2015). Although political commitments, international agreements, processes and programs have highlighted the importance of cultural landscapes, including wood-pastures, as a foundation for sustainable rural development that maintains multiple goods, services and values (CE 2000; MCPFE 2003; EC 2013; ENRD 2014), such valuable landscapes are still declining which pose great uncertainty for the conservation of the biodiversity and cultural values they uphold (Plieninger et al. 2015). This calls for finding alternatives for the restoration and conservation of wood-pastures, for which rewilding interventions could be a potential solution.

**Fig. 3:**
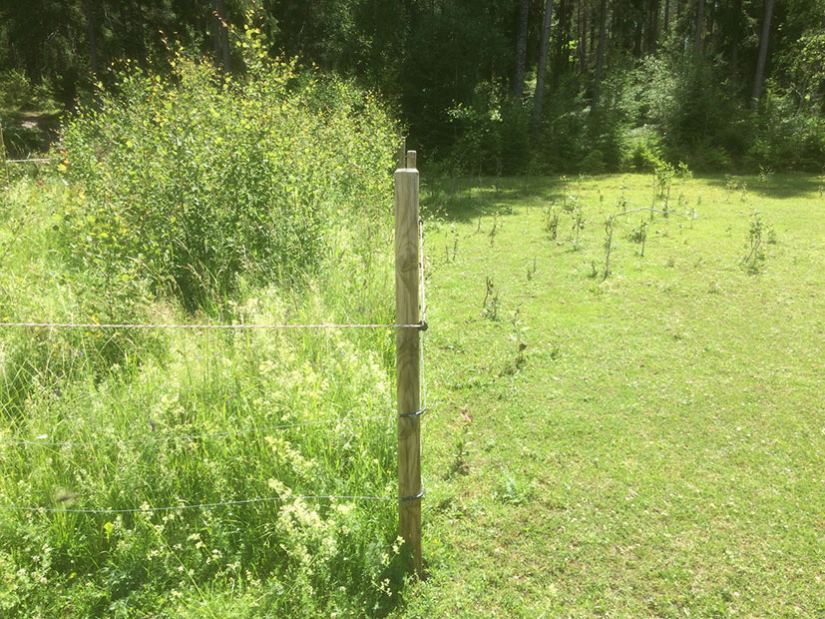
Effects of grazing and browsing on experimental and control areas at Krusenberg estate in 2016 (after 3-year experiment)

Our results show that horses significantly reduced understory vegetation densities in experimental conditions compared to controls (see “Results”; Figs. S1, S2). They additionally modified the vegetation structure and limited tree colonization of grasslands (Table 1; Figs. 2, 3). In this line, Kuiters and Slim (2003) reported clear effects of introducing Iceland ponies to maintain open grasslands in a nature reserve in the Dutch-Belgian border after 27 years of abandonment of former arable fields. Oldén et al. (2017) also reported clear effects of grazing on tree regeneration, in particular on preferred broadleaved tree species. These results support our findings (see Fig. 3) where horses radically limited tree colonization of grasslands. Kuiters and Slim (2003) additionally assessed habitat selection and habitat use, highlighting a clear increase of woodland habitat use from early autumn. This might be the result of phenology and preferred resource depletion (grasslands) forcing horses to feed elsewhere. In higher latitudes, such an effect should be even greater and may therefore explain the high browsing pressure of horses on trees detected in our experimental conditions.

Horses additionally affected tree species composition via selective browsing, which resulted in four times higher probability of ash being browsed compared to pine (see Table 2 and Appendix S1 for additional discussion on browsing effects on forest structure and composition by wild ungulates). This information may guide future biodiversity-oriented management efforts for abandoned wood-pastures and semi-natural grasslands in a rewilding context. In other studies, the regeneration of woodlands have been reported to be facilitated by associational resistance mechanisms, where browse-sensitive species like oak are protected by certain shrub species (Olff et al. 1999; Kuiters and Slim 2003; Bakker et al. 2004; Van Uytvanck et al. 2008). Indeed, Vera (2000) stated that the colonization of thorny shrub species is often stimulated by grazers due to the creation of local disturbances and vegetation gaps. In our study, blackthorn was abundant in the edge zone between forest and grasslands which might have allowed for mechanisms of associational resistance; although such mechanisms were not accounted for in designing the experiment, they might well explain our results in relation to oak (no reduction in height in experimental conditions compared to controls given the observed high browsing pressure (53%) levels). The effects of richer herbivore communities on forest and wood-pasture ecosystems may be modulated by habitat productivity and herbivore size (Bakker et al. 2006, 2016); however, knowledge on how vegetation structural and compositional changes occur is still limited. Our experiment provide detailed evidence based on tree preference. This calls for designing longer scientific rewilding experiments with different herbivore stocking rates, digestive physiology and feeding guilds, at diverse habitat productivity gradients to upscale and fully understand the potential effect of such interventions to advance rewilding science (Garrido et al. 2019). Richer herbivore communities, including large herbivore grazers, promote more open landscape conditions as well as plant compositional and structural changes (Bakker et al. 2016), which might be necessary to maintain mosaic landscapes and wood-pasture structures (Vera 2000; Vera et al. 2006). The ecological impact of richer mammalian herbivore communities may also depend on the density and diversity of the herbivore community as well as foraging habits, i.e., grazing, browsing or mix-feeding (Owen-Smith 1987), and seed (Griffiths et al. 2011) and nutrient dispersal (Doughty et al. 2016). Indeed, the most common rewilding species in Europe, i.e., European bison (*Bison bonasus* L.), and rustic cattle and horse breeds, have functionally diverse diets (Cromsigt et al. 2018a) and should thus be taken into account when designing future rewilding initiatives.

Our results support the potential of rewilding to restore abandoned wood-pastures, by promoting a more open vegetation structure and limiting the ability of pioneer tree species to successfully colonize grassland-dominated areas (Fig. 3). Rewilding interventions may go beyond the above-mentioned effects and even enhance grassland ecosystem functioning and biodiversity. In a parallel study from the same rewilding experiment, Garrido et al. (2019) noted that horse grazing enhanced the functional composition of grasslands, mitigated plant species declines, in particular bee-pollinated plants, and boosted pollinator habitat use. Ringmark et al. (2019) also reported that horse grazing diversified pasture chemical composition and enhanced its nutritional value. Hence, horse grazing may dually contribute to the restoration of wood-pastures and associated biodiversity and ecosystem functioning. This implies that the reintroduction of ecologically functional substitutes of extinct wild herbivores can have profound effects on ecosystems and promote trophic cascade effects that may restore lost processes and functions important to maintain biodiversity and the ecological integrity of herbivore-dependent ecosystems (Garrido et al. 2019). However, this may not materialize due to strict national and international policy. For instance, animal welfare, public health, and legal liability policies may impede or even undermine the development of future rewilding efforts. In Spain (Galicia region) incentives to comply with the European Commission Regulation EC 504/2008 on methods for identification of *equidae* and the EC 1774/2002 on food availability for necrophagous birds may threaten the fragile equilibrium between traditional free-ranging horse husbandry, heathland and wolf conservation (López-Bao et al. 2013). Similarly in Sweden, current regulations stipulate that horses have to be checked on daily basis, be provided with shelter during wintertime, and be protected from, e.g., predation and starvation. In short, EU and national policies may limit or even jeopardize future rewilding efforts in a situation that urgently calls for finding alternative solutions for the conservation of valuable grazing-dependent agricultural landscapes. In the Netherlands for instance, horses to be introduced in the Oostvaardersplassen rewilding area were re-classified as wild animals and thus no longer falling under livestock legislation (Naundrup and Svenning 2015, and references therein). Yet, this may not materialize without a necessary paradigm and political change to promote rewilding interventions (Jepson et al. 2018) and thus advance empirical rewilding science. In EU the revision of environmental policies and legislation particularly relevant for the future implementation of rewilding projects are the Birds and Habitats Directives and the Common Agricultural Policy. These directives were conceptualized and debated four decades back and were based on the preservation of particular species assemblages and habitat types (Jepson 2016). To aid the integration of rewilding into policy, more ecological, quantitative, and data-driven research is required (Pettorelli et al. 2018). Thus, certain key research areas have been recently identified (Pettorelli et al. 2018): (1) target setting and implementation to aid how to best choose management actions necessary to reach specific targets while maximizing biodiversity; (2) risk assessment to evaluate appropriate risk management and aid policy despite uncertainty; (3) economic costs and associated benefit assessment to facilitate cost-effective decision-making; (4) identification and characterization of social impacts to better understand the potential socio-economic effects of rewilding projects; and (5) monitoring and evaluation to ensure project trajectory and targets remain desirable for a given social–ecological system. Up-scaling rewilding models might however additionally require innovations in conservation finances and business models (Jepson et al. 2018).

To conclude, rewilding projects designed as scientific experiments are currently lacking (Svenning et al. 2016). Yet rewilding-inspired initiatives are being implemented in Europe, they lack scientifically designed and intensive monitoring to elucidate the ecological impacts exerted by the rewilding species on ecosystems. We present the first scientific empirical evidence of the effect of rewilding with horses to restore wood-pastures, and provide additional quantitative browsing pressure and tree selection estimates to inform future conservation-oriented management programs. Rustic horse breeds (as Gotland Russ in the present study) have the potential to survive year-round without supplementary feeding and are therefore suitable rewilding candidates. They can additionally affect the forest composition and structure as well as prevent tree colonization of grasslands with positive effects for biodiversity and wood-pasture restoration. The reduction of understory vegetation density and vegetation continuity might become a valuable ecosystem service by reducing fuel and thus preventing wildfire occurrence (Johnson et al. 2018). The rampant abandonment of European agricultural landscapes may offer great opportunities for rewilding (Navarro and Pereira 2015) while new frameworks to design and evaluate their effects are just being developed (Perino et al. 2019).

## Electronic supplementary material

Below is the link to the electronic supplementary material.

Supplementary material 1 (PDF 287 kb)

## Footnotes

1 Trees on grassland-dominated areas were excluded as these trees after 3-year experimental time were totally consumed (see Fig. 3).
