# Supplementary material for: Experimental rewilding may restore abandoned wood-pastures if policy allows
Source: Ambio. 2020 Mar 9;50(1):101–12. doi: 10.1007/s13280-020-01320-0 (PMC7708577; doi:10.1007/s13280-020-01320-0)
Supplement: Supplementary file 1 — Supplementary material 1 (PDF 287 kb) [file 13280_2020_1320_MOESM1_ESM.pdf]

Ambio

Electronic Supplementary Material

This supplementary material has not been peer reviewed

Title: **Experimental rewilding may restore abandoned wood-pastures if policy allows**

Authors: Pablo Garrido, Lars Edenius, Grzegorz Mikusiński, Anna Skarin, Anna Jansson, Carl-Gustaf Thulin

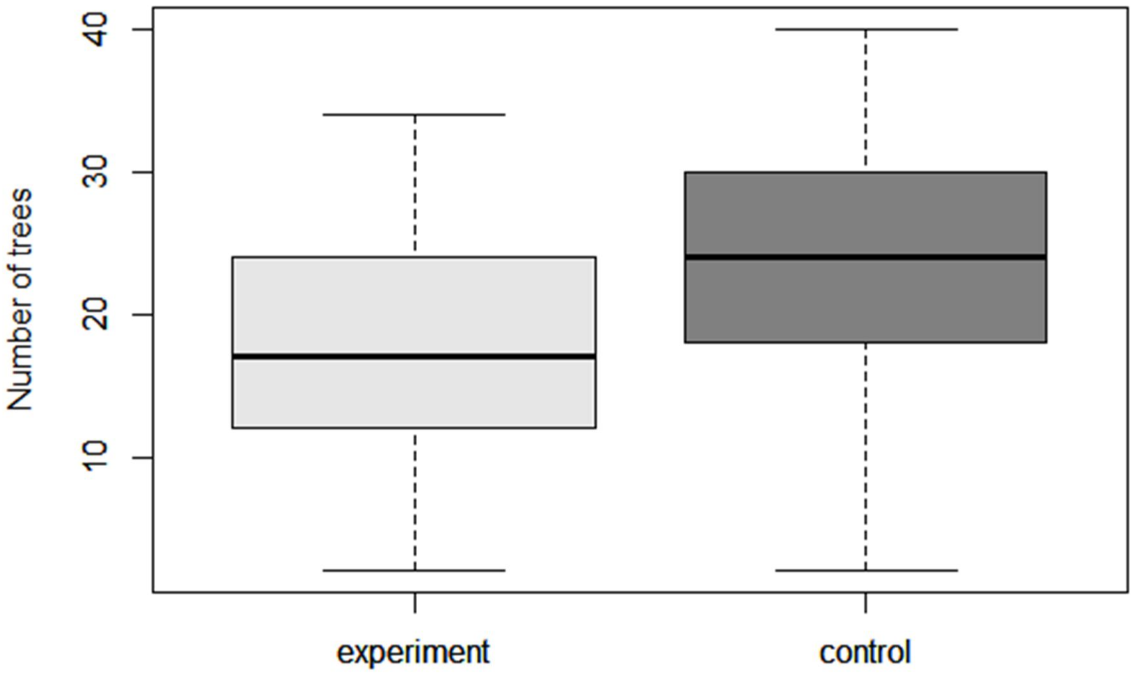

Figure S1. Total number of trees (y axis) in experimental vs control plots (x axis) after 3 years of experimental treatment. The solid black line in the box indicates the sample median. The horizontal edges of the box represent the interquartile range of the sample (i.e., 50% of the data).

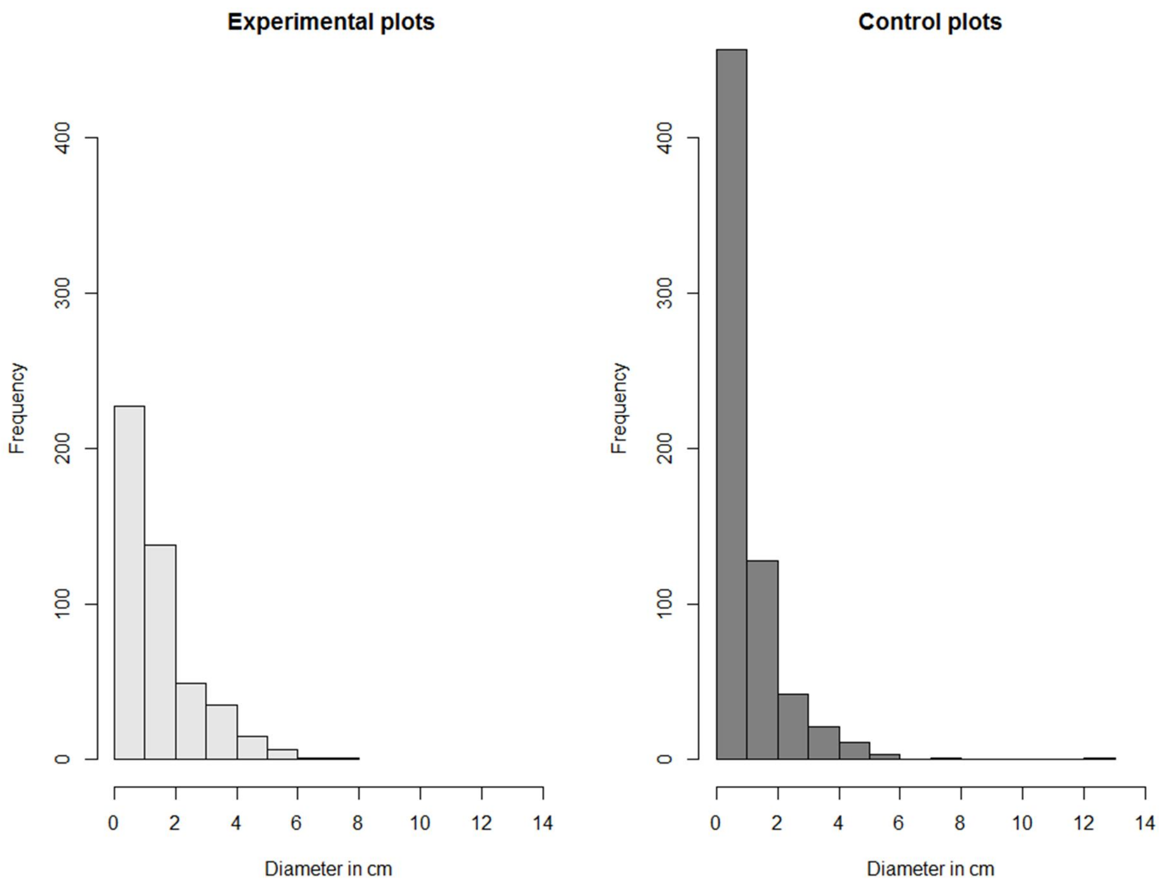

Figure S2. Histogram representing the number of trees per diameter class in experimental and control plots. Mature trees, i.e., trees higher than 5 m, are excluded.

Table S1. Total number of tree species surveyed and their frequency of occurrence.

Scientific name	English name	N trees (forest)	N trees (grassland)
Acer platanoides	Norway maple	5	0
Betula alba	Downy birch	3	0
Betula pendula	Silver birch	96	39
Coryllus avellana	Common hazel	1	0
Fraxinus excelsior	European ash	58	0
Juniperus communis	Juniper	4	0
Picea abies	Norway spruce	193	32
Pinus sylvestris	Scots pine	41	9
Populus tremula	Aspen	351	146
Prunus padus	Hackberry tree	8	1
Prunus spinosa	Blackthorn	112	36
Quercus robur	Pedunculate oak	90	0
Salix spp.	Sallow	73	28
Sorbus aucuparia	Rowan	95	0
Sorbus intermedia	Swedish whitebeam	5	0

*Trees higher than 5 m amounted 89; 5 birch, 1 European ash, 31 Norway spruce, 26 Scots pine, 22 aspen, 2 pedunculate oak and, 2 rowan species (not included in the table).

Discussion

We found clear differences in height/diameter ratios for trees living in experimental conditions (presence of large herbivores) in comparison to controls. In particular, for a given diameter, seedlings and saplings of *B. pendula*, *P.tremula*, *P. spinosa*, *Salix* spp., *S. aucuparia* and *F. excelsior* were lower at experimental conditions than in controls (Table 1, Figure 2). The absence of such relation in *P. sylvestris* is likely due to its low browsing pressure (Table 2). For *P. abies* however, no browsed twig was recorded and it was thus likely avoided (Irving 2001). For *Q. robur* recruitment may be prevented by browsing (Kuiters and Slim 2002, Götmark et al. 2005), although resistance to browsing has also been reported (Götmark and Kiffer 2014). This in combination to *Q. robur* associational resistance mechanisms (Bakker et al. 2004) may explain the absence of differences in height/diameter ratios despite the observed high browsing pressure levels (53%).

The total number of seedlings and saplings were also significantly lower in experimental conditions compared to controls. Likewise, a reduction of understory foliage proportion in woodlands with high concentration of deer has been detected both through field studies (e.g., Côté et al. 2004) and more recently by terrestrial laser scanning (Eichhorn et al. 2017). Seedling density has also been reported to decrease as deer density increase (Gill and Morgan 2010); pattern commonly observed in previous investigations (Gill 1992a, b, Gill 2006, Ward et al. 2008). Reductions of the understory structural complexity has also been described in response to high deer numbers (Corney et al. 2008, Martin et al. 2010), as well as altered tree size distributions (Peltzer et al. 2014). The magnitude of the effect however, may be modulated by the preference of deer for certain tree species (Månsson et al. 2007), the relative forage quality (Pollock et al. 2005) and availability (Hörnberg 2001, Bergqvist et al. 2014), plant resistance mechanism to herbivory (Gill 2006), and previous history of browsing (Palmer and Truscott 2003). Our results show that the experimental herbivore affected tree species composition via selective browsing. Indeed a decreasing browsing pressure from European ash to rowan, oak, sallow, blackthorn, aspen, silver birch and scots pine was found, ranging from 71% of browsed twigs in ash to 19% for pine. This resulted in a four times higher probability of ash of being browsed compared to pine. Similar results were reported by Månsson et al. (2007) although moose

exerted a much higher selectivity estimates for preferred tree species. A consequence of a sustained browsing pressure on preferred tree species can lead to plant compositional changes and the dominance of browsing resistant species (Gill 1992a, Gill and Beardall 2001, Côté et al. 2004, Gill 2006, White 2012, Holm et al. 2013). For instance, Tilghman (1989) reported for white-tailed deer (*Odocoileus virginianus*) a decline of browse-sensitive species with increasing deer density. More recently, decades of sustained browsing pressure have been shown to limit palatable species development and shifts of dominance towards non-preferred species such as *Picea glauca* in North America (White 2012). In Japan intensive sika deer (*Cervus nippon*) browsing has altered the forest structure and composition and favored unpalatable shade-intolerant plant species (Takatsuki 2009). Similarly, in the Bialowieza Primeval Forest in Poland, where a full assemblage of native large herbivores still persist (including European bison, red deer, roe deer, wild boar and moose), it has been shown that herbaceous vegetation cover was the main factor determining the number of seedlings, that soil fertility was positively associated to sapling ≤ 50 cm density, and that herbivory modulated tree recruitment rate of trees > 50 cm (Kuijper et al. 2010). Additionally the majority of herbivores preferred forest gaps for feeding (Kuijper et al. 2009).

References

Bakker, E. S., H. Olff, C. Vandenberghe, K. De Maeyer, R. Smit, J. M. Gleichman, and F. W. M. Vera. 2004. Ecological anachronisms in the recruitment of temperate light-demanding tree species in wooded pastures. Journal of Applied Ecology **41**:571-582.

Bergqvist, G., R. Bergström, and M. Wallgren. 2014. Recent browsing damage by moose on Scots pine, birch and aspen in young commercial forests–effects of forage availability, moose population density and site productivity. Silva Fennica **48**:1-13.

Corney, P., K. Kirby, M. Le Duc, S. Smart, H. McAllister, and R. Marrs. 2008. Changes in the field-layer of Wytham Woods–assessment of the impacts of a range of environmental factors controlling change. Journal of Vegetation Science **19**:287-298.

Côté, S. D., T. P. Rooney, J.-P. Tremblay, C. Dussault, and D. M. Waller. 2004. Ecological impacts of deer overabundance. Annual Review of Ecology, Evolution and Systematics **35**:113-147.

Eichhorn, M. P., J. Ryding, M. J. Smith, R. Gill, G. M. Siriwardena, and R. J. Fuller. 2017. Effects of deer on woodland structure revealed through terrestrial laser scanning. Journal of Applied Ecology **54**:1615–1626.

Gill, R. M. A. 1992a. A Review of Damage by Mammals in North Temperate Forests: 1. Deer. Forestry: An International Journal of Forest Research **65**:145-169.

Gill, R. M. A. 1992b. A Review of Damage by Mammals in North Temperate Forests: 3. Impact on Trees and Forests. Forestry: An International Journal of Forest Research **65**:363-388.

Gill, R. M. A. 2006. The influence of large herbivores on tree recruitment and forest dynamics. Pages 170-202 *in* K. Danell, R. Bergström, P. Duncan, and J. Pastor, editors. Large Herbivore Ecology, Ecosystem Dynamics and Conservation. Cambridge University Press, New York.

Gill, R. M. A., and V. Beardall. 2001. The impact of deer on woodlands: the effects of browsing and seed dispersal on vegetation structure and composition. Forestry **74**:209-218.

Gill, R. M. A., and G. Morgan. 2010. The effects of varying deer density on natural regeneration in woodlands in lowland Britain. Forestry: An International Journal of Forest Research **83**:53-63.

Götmark, F., Å. Berglund, and K. Wiklander. 2005. Browsing damage on broadleaved trees in semi-natural temperate forest in Sweden, with a focus on oak regeneration. Scandinavian Journal of Forest Research **20**:223-234.

Götmark, F., and C. Kiffer. 2014. Regeneration of oaks (*Quercus robur*/*Q. petraea*) and three other tree species during long-term succession after catastrophic disturbance (windthrow). Plant Ecology **215**:1067-1080.

Holm, J. A., J. R. Thompson, W. J. McShea, and N. A. Bourg. 2013. Interactive effects of chronic deer browsing and canopy gap disturbance on forest successional dynamics. Ecosphere **4**:1-23.

Hörnberg, S. 2001. The relationship between moose (Alces alces) browsing utilisation and the occurrence of different forage species in Sweden. Forest Ecology and Management **149**:91-102.

Irving, B. 2001. The impacts of horse grazing on conifer regeneration in west-central Alberta. University of Alberta.

Kuijper, D. P., J. Cromsigt, M. Churski, B. Adam, B. Jędrzejewska, and W. Jędrzejewski. 2009. Do ungulates preferentially feed in forest gaps in European temperate forest? Forest Ecology and Management **258**:1528-1535.

Kuijper, D. P., J. P. Cromsigt, B. Jędrzejewska, S. Miścicki, M. Churski, W. Jędrzejewski, and I. Kweczlich. 2010. Bottom-up versus top-down control of tree regeneration in the Białowieża Primeval Forest, Poland. Journal of Ecology **98**:888-899.

Kuiters, A. T., and P. A. Slim. 2002. Regeneration of mixed deciduous forest in a Dutch forest-heathland, following a reduction of ungulate densities. Biological Conservation **105**:65-74.

Martin, J.-L., S. A. Stockton, S. Allombert, and A. J. Gaston. 2010. Top-down and bottom-up consequences of unchecked ungulate browsing on plant and animal diversity in temperate forests: lessons from a deer introduction. Biological Invasions **12**:353-371.

Månsson, J., C. Kalén, P. Kjellander, H. Andrén, and H. Smith. 2007. Quantitative estimates of tree species selectivity by moose (Alces alces) in a forest landscape. Scandinavian Journal of Forest Research **22**:407-414.

Palmer, S. C. F., and A. M. Truscott. 2003. Browsing by deer on naturally regenerating Scots pine (Pinus sylvestris L.) and its effects on sapling growth. Forest Ecology and Management **182**:31-47.

Peltzer, D. A., R. B. Allen, P. J. Bellingham, S. J. Richardson, E. F. Wright, P. I. Knightbridge, and N. W. Mason. 2014. Disentangling drivers of tree population size distributions. Forest Ecology and Management **331**:165-179.

Pollock, M. L., J. M. Milner, A. Waterhouse, J. P. Holland, and C. J. Legg. 2005. Impacts of livestock in regenerating upland birch woodlands in Scotland. Biological Conservation **123**:443-452.

Takatsuki, S. 2009. Effects of sika deer on vegetation in Japan: A review. Biological Conservation **142**:1922-1929.

Tilghman, N. G. 1989. Impacts of White-Tailed Deer on Forest Regeneration in Northwestern Pennsylvania. The Journal of wildlife management **53**:524-532.

Ward, A. I., P. C. White, N. J. Walker, and C. H. Critchley. 2008. Conifer leader browsing by roe deer in English upland forests: Effects of deer density and understorey vegetation. Forest Ecology and Management **256**:1333-1338.

White, M. A. 2012. Long-term effects of deer browsing: Composition, structure and productivity in a northeastern Minnesota old-growth forest. Forest Ecology and Management **269**:222-228.
